# Alleviation of Photoinhibition by Co-ordination of Chlororespiration and Cyclic Electron Flow Mediated by NDH under Heat Stressed Condition in Tobacco

**DOI:** 10.3389/fpls.2016.00285

**Published:** 2016-03-30

**Authors:** Qinghua Li, Zheng-Ju Yao, Hualing Mi

**Affiliations:** National Key Laboratory of Plant Molecular Genetics and Photosynthesis, Institute of Plant Physiology and Ecology, Shanghai Institutes for Biological Sciences, Chinese Academy of SciencesShanghai, China

**Keywords:** NAD(P)H dehydrogenase, cyclic electron transport, chlororespiration, plastid terminal oxidase, heat stress, tobacco

## Abstract

With increase of temperature, *F*_o_ gradually rose in both WT and the mutant inactivated in the type 1 NAD(P)H dehydrogenase (NDH), a double mutant disrupted the genes of *ndhJ* and *ndhK* (Δ*ndh*JK) or a triple mutant disrupted the genes of *ndhC, ndhJ*, and *ndhK* (Δ*ndhCJK*). The temperature threshold of *F_o_* rise was about 3–5°C lower in the mutants than in WT, indicating Δ*ndh*JK and Δ*ndhCJK* were more sensitive to elevated temperature. The *F*_o_ rise after the threshold was slower and the reached maximal level was lower in the mutants than in WT, implying the chlororespiratory pathway was suppressed when NDH was inactivated. Meanwhile, the maximum quantum efficiency of photosystem II (PS II) (*F*_v_*/F*_m_) decreased to a similar extent below 50°C in WT and mutants. However, the decline was sharper in WT when temperature rose above 55°C, indicating a down regulation of PS II photochemical activity by the chlororespiratory pathway in response to elevated temperature. On the other hand, in the presence of *n*-propyl gallate, an inhibitor of plastid terminal oxidase (PTOX), the less evident increase in *F*_o_ while the more decrease in *F*_v_*/F*_m_ in Δ*ndhCJK* than in WT after incubation at 50°C for 6 h suggest the increased sensitivity to heat stress when both NDH and chlororespiratory pathways are suppressed. Moreover, the net photosynthetic rate and photo-efficiency decreased more significantly in Δ*ndh*JK than in WT under the heat stressed conditions. Compared to the light-oxidation of P700, the difference in the dark-reduction of P700^+^ between WT and *ndh*JK disruptant was much less under the heat stressed conditions, implying significantly enhanced cyclic electron flow in light and the competition for electron from PQ between PTOX and photosystem I in the dark at the elevated temperature. Heat-stimulated expression of both NdhK and PTOX significantly increased in WT, while the expression of PTOX was less in Δ*ndh*JK than in WT. Meanwhile, the amount of active form of Rubisco activase decreased much more in the mutant. The results suggest that chlororespiration and cyclic electron flow mediated by NDH may coordinate to alleviate the over-reduction of stroma, thus to keep operation of CO_2_ assimilation at certain extent under heat stress condition.

## Introduction

The concept of chlororespiration was used to describe the respiratory electron transport pathway within the chloroplast (Bennoun, 1982). Different from mitochondrial respiration, chlororespiration links to the photosynthetic electron transport chain by sharing the PQ pool and affecting its redox state in green algae (Bennoun, 1982, 1983) and in higher plants (Garab et al., 1989) in darkness. According to the model of chlororespiration (Bennoun, 1982; Peltier and Cournac, 2002), PQ connects the electron transport from NAD(P)H to O_2_ by means of non-photochemical reduction of PQ by NAD(P)H and subsequent oxidation by a putative terminal oxidase, resulting in proton transfer from the stroma to the lumen of thylakoid membranes. Further evidence supporting the operation of chlororespiration includes: (1) genes with high sequence homology to those encoding the subunits of mitochondrial complex I have been found in chloroplasts (Ohyama et al., 1986; Shinozaki et al., 1986); the *ndh* genes encode at least 15 subunits (Ndh-A-O), among which NdhA–NdhK are plastid-encoded and the rest (NdhL–NdhO) are nuclear-encoded (Ifuku et al., 2011), and (2) a protein designated PTOX with sequence homology to alternative oxidases of plant mitochondria has been identified in chloroplasts of *Arabidopsis thaliana* (Carol et al., 1999; Wu et al., 1999). The involvement of PTOX in PQ oxidation using molecular O_2_ as a terminal electron acceptor has been demonstrated by analysis of AtPTOX-overexpressing transgenic tobacco plants (Joet et al., 2002b).

Except in the high mountain plant species *Ranunculus glacialis* (Streb et al., 2005), the amount of PTOX is minor in many plant species so far examined. Thus, the capacity of PTOX-dependent consumption of excess electrons is considered to be low (Ort and Baker, 2002; Peltier and Cournac, 2002). However, chlororespiration becomes obvious under stress conditions. Many works have shown that the cyclic electron flow around PS I mediated by NDH functions in protecting plants against environmental stresses such as high light (Martin et al., 1996; Endo et al., 1999), elevated or low temperature (Wang et al., 2006) and water stress (Horvath et al., 2000). On the other hand, based on the high abundance of PTOX in alpine plant species acclimated to high light and its decline during deacclimation, it has been suggested that chlororespiration is the second major electron sink in *R. glacialis* with oxygen as final acceptor (Streb et al., 2005). Evidence shows that chlororespiration also protects plant against environmental stresses such as heat or high light conditions (Quiles, 2006; Diaz et al., 2007), chilling stress (Ivanov et al., 2012; Segura and Quiles, 2015), drought stress (Ibanez et al., 2010; Paredes and Jose Quiles, 2013). Both the cyclic electron flow around PS I and chlororespiration function during photosynthesis under changing environmental conditions (Rumeau et al., 2007). However, how the cyclic electron flow around PS I and chlororespiration are coordinated in their protective roles still remains to be further investigated.

In this work, we compared the changes in *F*_o_ upon increase of temperature, the photosynthetic capacities, and the expression level of PTOX and Rubisco activase between wild type (WT) and the NDH inactivated mutants, a double mutant of *ndh*J and *ndh*K (Δ*ndh*JK) or a triple mutant of *ndh*C, *ndhJ*, and *ndh*K (Δ*ndhC*JK) of tobacco at elevated temperature. Our results suggest that both the chlororespiration and cyclic electron flow mediated by NDH are coordinated to alleviate photoinhibition during heat stress.

## Materials and Methods

### Growth Conditions of Plants

The homoplasmic Δ*ndh*JK or Δ*ndhC*JK (*Nicotiana tabacum* cv. *Xanthi*) plants, in which the chloroplastic *ndhJ* and *ndhK* genes (Takabayashi et al., 2001) or *ndhC, ndhJ*, and *ndhK* genes (Takabayashi et al., 2002) were insertionally inactivated, were cultivated along with WT in the phytotron with a rhythm of 14 h light at 25°C and 10 h dark at 20°C, a humidity of 40%, and a light intensity of 200 μmol m^-2^ s^-1^. For the experiments, 4–5 week-old plants were used.

### Heat Treatment

Plants grown in pots were transferred into a chamber (LRH-250A-G incubator, Zhujiang China) with a set temperature at 50°C, light intensity of 100 μmol m^-2^ s^-1^, and 70% humidity. Control plants were kept at 28°C, with other conditions identical to the heat treatments. To ensure no significant water loss during heat treatment, plant pots with holes in the bottom were put in a tray containing water of 2 cm-deep and sprayed with 50°C water on a regular basis. The *F*_o_ changes upon increase in temperature were measured as described in a previous study (Pastenes and Horton, 1999); the attached first trifoliate leaf was placed on the surface of a thermostated cuvette that was connected to circulating water bath with increasing temperature from 20 to 60°C.

### Chlorophyll Fluorescence and the Redox State of P700

Chlorophyll fluorescence and the redox state of P700 were measured with PAM chlorophyll fluorometer (Walz, Effeltrich, Germany) with an emitter-detector (ED-101 US) for chlorophyll fluorescence and another (ED-P700DW-E) for P700 absorbance changes monitored by the absorbance at 810–830 nm. The fluorometer setup was as described as by the previous studies (Schreiber et al., 1986) and (Klughammer and Schreiber, 1998). The dark level chlorophyll fluorescence (*F*_o_) was measured with a weak, modulated red light (650 nm, 0.09 μmol photons m^-2^ s^-1^). Maximum chlorophyll fluorescence (*F*_m_) was measured after a 0.8 s pulse of saturated white light. Maximum quantum efficiency of PS II was determined by *F*_v_*/F*_m_. ΦPSII, the photochemical efficiency of PSII, was calculated as (F′_m_ - F)/F′_m_; qP and qN were calculated as (F′_m_ - F)/(F′_m_ - F′_o_) and 1 - (F′_m_ - F′_o_)/(F_m_ - F_o_), respectively, after steady-state photosynthesis was reached (15 min of light induction together with saturating pulses of 0.8 s every 30 s), and *F*_m_ here was determined before stress. The halftime of the oxidation of P700 was determined after reaching a steady state level of P700^+^ by illumination with FR (>705 nm, 5.2 μmol m^-2^ s^-1^) and that of re-reduction of the P700^+^ was determined after a 6 s illumination with FR.

### Net Photosynthetic Rate

Net photosynthetic rate was measured with a portable photosynthesis system (model 6400, Li-Cor Inc.). The measurement was performed on attached leaves at 28°C before and after heat treatment for 6 h with a light intensity of 1000 μmol photons m^-2^ s^-1^ PAR, and 350 ppm CO_2_ in the sample chamber. Data were collected 2 min after leaves being placed in the sample chamber.

### Isolation of Chloroplast Extracts

Intact chloroplasts were isolated from freshly harvested leaves with a method described by a previous study (Wang et al., 2006). The chloroplasts were osmotically ruptured in a medium containing 50 mM Tris-HCl (pH 7.5), and then centrifuged at 4000 *g* for 10 min at 4°C. The supernatant was collected as the stromal fraction, the pellet was washed twice with medium containing 0.4 M sucrose, 10 mM NaCl, and 50 mM Tris–HCl (pH 7.6), and re-suspended in the medium as sample of thylakoid membranes. Samples were stored at -80°C until use. Chlorophyll content was determined according (Porra et al., 1989).

### Gel Electrophoresis and Western Blotting

Denatured proteins were separated by SDS-PAGE in a 15% polyacrylamide gel according to (Laemmli, 1970). Proteins in the gel were electrically transferred to a nitrocellulose membrane for Western blotting analysis using an ECL immunoblotting kit (Amersham). Protein concentration was determined by the method described by Bradford (1976).

The antibody raised against the K subunit of NDH (NdhK) from pea was a gift from Dr. J. M. Arizmendi, Eustal Herriko Unibertsitatea, Spain. Antibodies against Rubisco activase and 33 kDa protein of PS II core complex were kindly provided by Dr GY Chen and Prof. C Xu, respectively, from Institute of Plant Physiology and Ecology, Shanghai Institutes for Biological Sciences, Chinese Academy of Sciences. PTOX antibody was a gift from Dr. Kuntz Marcel, Université Joseph Fourier and CNRS (Plastes et Différenciation Cellulaire, UMR 5575), Grenoble, France.

### Inhibitor Treatment of the Attached Leaf

Four-week-old plants were used for the experiments. Fully expanded mature leaves were chosen for inhibitor treatment. The symmetrical areas of the leaves separated by the midrib were infiltrated with the solution of 4 mM *n*-propyl gallate (TCI company, Japan) or with the same concentration of ethanol diluted in distilled water as control. Infiltration was achieved by pressing 100 μl of *n*-propyl gallate or ethanol solution into the abaxial side of the leaves using the blunt end of a 1 ml syringe as described (Wu et al., 2011). The infiltrated spot was around 4 cm away from the midrib and the total infiltrated area covered approximately 3–5 cm^2^.

## Results

### Effect of Raising Temperature on Apparent Dark Level of Chlorophyll Fluorescence

**FIGURE 1:**
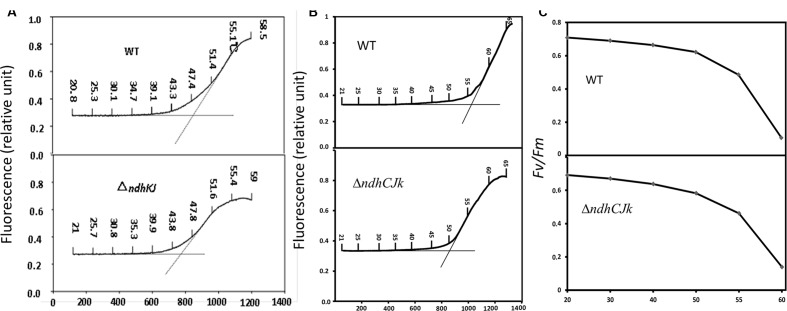
**Changes in *F*_o_ (relative units) **(A,B)** and *F*_v_*/F*_m_**(C)** upon increases in temperature with time in WT, Δ*ndh*KJ and Δ*ndhC*KJ.** Dotted vertical lines represent the threshold of *F*_o_ rise.

Heat stress has been suggested to lead to an increase in the dark level of chlorophyll fluorescence *F*_o_ (Schreiber and Berry, 1977) due to the detachment of LHC II from PS II complex and the inactivation of PS II photochemical reaction (Schreiber and Armond, 1978), or the reduction of Q_A_ in the dark (Yamane et al., 2000). The temperature threshold of *F*_o_ rise was used to estimate the resistance to high temperature in two bean varieties (Pastenes and Horton, 1999). With increased leaf temperature from 20 to 60°C, the thresholds were about 45°C in Δ*ndhJK* and 48°C in WT, respectively. The temperature threshold of *F*_o_ rise was about 3°C lower in the mutant than in WT (**Figure 1A**). In another comparison, the thresholds were 50°C in Δ*ndhCJK* and 57°C in WT, respectively. The temperature threshold of *F*_o_ rise was about 7°C lower in the mutant than in WT (**Figure 1B**). These results indicate that tobacco plant is more sensitive to the elevated temperature when *ndhC*JK genes were defective. After the temperature threshold, the *F*_o_ rose more slowly and the reached maximal level was lower in the mutants than in WT. The result implies that the chlororespiratory pathway was suppressed when NDH was inactivated.

### Effect of Raising Temperature on PS II Photochemical Activity

*F*_v_*/F*_m_ is a chlorophyll fluorescence parameter used to evaluate the maximum or potential quantum efficiency of PS II (Genty et al., 1989). **Figure 1C** shows that with the increased temperature, the *F*_v_*/F*_m_ decreased to a similar extent until 50°C in WT and *ndh*CJK mutant. However, the decline was sharper in WT when temperature rose above 55°C, indicating a down regulation of PS II photochemical activity by the chlororespiraory pathway in response to the elevated temperature.

### Effect of an Inhibitor of Chloroespiration on *F*_o_ and *F*_v_/*F*_m_ after Adaption at the Elevated Temperature

**FIGURE 2:**
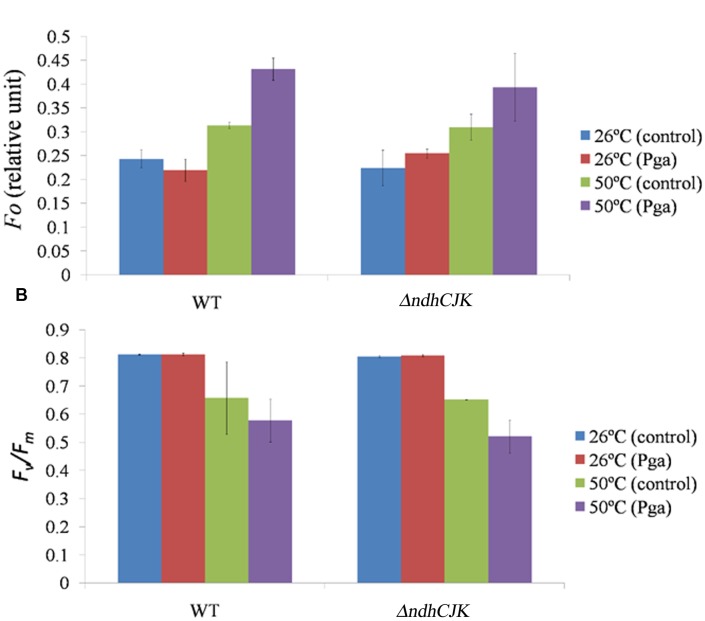
**Comparison of the chlorophyll parameters *F*_o_ level **(A)** and *F*_v_*/F*_m_**(B)** between WT and Δ*ndhC*KJ of tobacco plants before (at 26°C) and after heat treatment (at 50°C for 6 h).** The level of *F*_o_ was measured 15 min after dark incubation of a leaf by infiltration of 4 mM *n*-propyl gallate (Pga) or the same concentration of ethanol (control), respectively. Data points represent the mean ± SE of three independent measurements.

To know how the change in *F*_o_ and *F*_v_*/F*_m_ after adaption at the elevated temperature, We further compared the *F*_o_ level between WT and Δ*ndhCJK* before and after incubation at 50°C for 6 h (**Figure 2A**). There was no significant difference in *F*_o_ level between WT and Δ*ndhC*KJ before the heat treatment. After incubation at 50°C for 6 h, the *F*_o_ levels increased by about 28% in WT and 38% in Δ*ndhC*KJ (**Figure 2A**), indicating more reduction of the intersystem chain in the mutant after adaptation at the elevated temperature. In the presence of *n*-propyl gallate, one of the inhibitors of PTOX (Joet et al., 2002b), the *F*_o_ level further increase by about 27% in Δ*ndhC*KJ and 38% in WT after the heat treatment for 6 h, suggesting the decrease in chlororespiratory pathway when NDH is inactivated. These results imply that the electrons are recycled from PS I reduced side through NDH to PQ, and finally transferred to molecular oxygen via PTOX, thereby alleviates the over reduction of intersystem chain.

### Parameters of Chlorophyll Fluorescence

**FIGURE 3:**
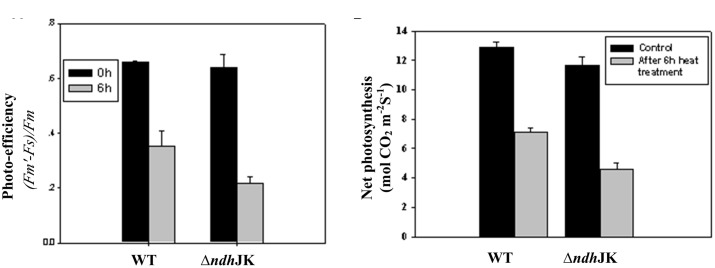
**The photo-efficiency of PS II **(A)** and the net photosynthetic rate **(B)** of WT and Δ*ndh*JK.** Measurements were done on attached leaves at 28°C (before) or after 6 h heat stress treatment (50°C) with a light intensity of 1000 μmol photons m^-2^ s^-1^ PAR, 350 ppm CO_2_ in the sample chamber. Recording was carried out 2 min after incubation of leaves in the sample chamber. Data points represent the mean ± SE of six replications.

Several parameters of chlorophyll fluorescence were used to evaluate photosynthesis reactions between the WT and the NDH mutants upon adaptation to an elevated temperature (50°C). Before the heat treatment, there was no difference in the parameter of *F*_v_*/F*_m_, which reflects PS II photochemical activity, but the value decreased to the similar extent in both Δ*ndhCJK* and WT after 6 h heat treatment (**Figure 2B**). In the presence of *n*-propyl gallate, *F*_v_*/F*_m_ further decreased by 20% in Δ*ndhCJK* and 12% in WT (**Figure 2B**), suggesting the increased sensitivity to the elevated temperature when both NDH pathway and chlororespiratory pathway were blocked. To investigate how the photo-efficiency of PS II changes under the heat stress condition, we compared another chlorophyll parameter (F′_m_ - F_s_)/F′_m_ between WT and Δ*ndh*JK. There was no significant difference in photo-efficiency of PS II between WT and Δ*ndh*JK before treatment, but the photo-efficiency in Δ*ndh*JK was only about 60% of that in WT (**Figure 3A**) after incubated at 50°C for 6 h. These results indicate that photo-inhibition was severe when the *NdhJ*, *NdhK* genes were both defective.

### Net Photosynthesis

We further studied how the photosynthetic capacity changes under the heat stress condition by comparing net photosynthesis between WT and Δ*ndh*JK. Before heat stress treatment, the net photosynthesis rates of Δ*ndh*JK and WT were almost identical (**Figure 3B**). However, after the exposure to 50°C for 6 h, the net photosynthesis dropped to 55.1% in the WT and 39.4% in Δ*ndh*JK. Meanwhile, the intracellular CO_2_ concentration (Ci) slightly increased in both WT and Δ*ndh*JK, and there was no detectable difference between them (data not shown). These results indicated that the decrease in net photosynthesis in Δ*ndh*JK was unlikely caused by the stomata closure, but by the inactivation of photosynthetic activity. Consistent with *F*_o_ analysis (**Figures 2** and **3**), net photosynthesis was more severely affected by the heat stress in Δ*ndh*JK than in WT. We therefore concluded that the photosynthetic machinery of Δ*ndh*JK is more sensitive to heat stress.

### Redox State of P700

The rate of electron donation to the intersystem chain from photoreductants accumulated in the stroma during illumination can be determined by monitoring redox changes in P700 (a pair of reaction center chlorophylls in PS I). This non-photochemical reduction of P700 is activated by heat stress (Bukhov et al., 1999). To evaluate the contribution of NDH to this electron flow, we compared the oxidation of P700 to P700^+^ induced by FR and the re-reduction of P700^+^ between WT and Δ*ndh*JK post FR treatment (**Table 1**). Before the heat stress treatment, the halftime of P700 oxidation in WT was 14% longer than that in Δ*ndh*JK. In contrast, the halftime of the P700^+^ re-reduction was about 18% shorter in WT than in Δ*ndh*JK. These results can be explained by the contribution of NDH in cyclic electron transport around PSI, similar to that in the triple mutant Δ*ndh*CJK (Wang et al., 2006). After the 6 h exposure to heat stress, the halftime of P700 oxidation increased in both WT (126%) and Δ*ndh*JK (55%), indicating that the NDH-dependent cyclic electron flow was remarkably enhanced in light. Meanwhile, the half time of P700^+^ re-reduction decreased in both WT (35%) and Δ*ndh*JK (27%), implying that NDH-dependent and independent electron flows to O_2_ via PTOX were also enhanced in the dark.

**Table 1:** Oxidation of P700 by far-red light (FR) and re-reduction of P700^+^ after turning off the FR in leaves of wild type (WT) and its *ndh*JK defective mutant (*ndh*JK mutant) of tobacco (*n* = 12) before and after heat treatment.

	Oxidation rate of P700, *t*_1/2_ (s)		Re-reduction of P700^+^, *t*_1/2_ (s)	
	28°C	50°C	28°C	50°C
WT	1.19 ± 0.07	2.70 ± 0.48	1.90 ± 0.31	1.23 ± 0.16
NdhJK mutant	1.02 ± 0.08	1.60 ± 0.11	2.33 ± 0.28	1.69 ± 0.19

*The oxidation of P700 induced by FR (>705 nm, 5.2 μmol m^-2^ s^-1^) and the re-reduction of the P700^+^ after turning off the FR were monitored by the absorbance at 810–830 nm. The measurements were carried out 2 min after incubation of the leaves on the surface of a thermostated cuvette at 28°C or at 50°C 6 h heat treatment. The halftime (*t*_1/2_) of oxidation of P700 was determined after reaching a steady state level of P700^+^ by FR and that of re-reduction of the P700^+^ was determined after turning off FR.*

### Expression of NdhK, PTOX, and Other Photosynthetic Proteins during Heat Stress

**FIGURE 4:**
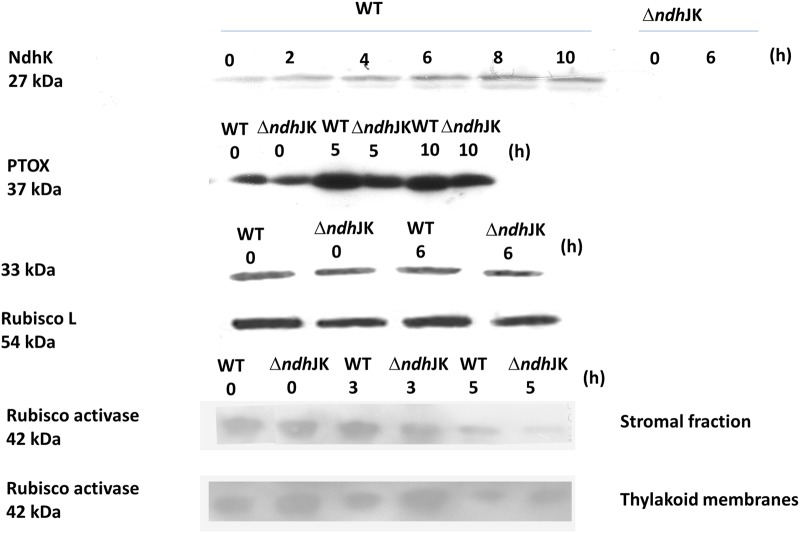
**Comparison of the expressions of NdhK, 33 kDa protein of a peripheral protein of PS II and Rubisco large subunit (Rubisco L), PTOX, and Rubisco activase between WT and Δ*ndh*JK.** The plants were transferred to the heat stress condition for indicated time before sampling. Each lane for NdhK and PTOX expression were loaded with 30 μg proteins, respectively, or for 33 kDa, Rubisco L and Rubisco activase with 15 μg proteins.

It has been demonstrated that the levels of NDH (Martin et al., 1996) and PTOX (Streb et al., 2005) are enhanced during photo-oxidative stress. To investigate how these proteins respond to heat stress conditions in WT and Δ*ndh*JK, Western blotting was performed using antibodies raised to against NdhK and PTOX. As control, antibodies against 33 kDa protein, a peripheral protein of PS II, Rubisco large subunit, and Rubisco activase were used. In WT, the protein level of NdhK evidently increased after incubation at 50°C (**Figure 4**). As expected, there was no detectable expression of NdhK in Δ*ndh*JK (**Figure 4**). The expression of PTOX was remarkably increased in both WT and Δ*ndh*JK, with more increase in WT than in the mutant (**Figure 4**). In contrast, little changes of 33 kDa protein and Rubisco large subunit were observed in both WT and Δ*ndh*JK before and after treatment (**Figure 4**). These results imply a physiological function of the chlororespiratory pathway to against heat stress via NDH. It was suggested that active form of Rubisco activase maintained soluble in stroma while its inactive form bound to thylakoid membranes due to the conformational change under high temperature stress condition (Rokka et al., 2001; Yang et al., 2005). With prolonged heat stressed time, the amount of Rubisco activase in stromal fraction (active form) decreased in both WT and Δ*ndh*JK, but to a greater extent in the mutant (**Figure 4**), indicating that the Rubisco activase was more sensitive to heat stress when NDH-dependent cyclic electron flow was inactivated. On the contrary, the amount of Rubisco activase in the thylakoid membrane (inactive form) was much higher in Δ*ndh*JK than in WT either before or after heat treatment (**Figure 4**). These results suggest that the confirmation change in Rubisco activase happens when NDH-dependent cyclic electron flow is inactivated and the heat-inactivated Rubisco activase does not bound to thylakoid membrane, while likely being degraded.

## Discussion

Chloroplast NDH has been suggested to function in protecting plants against stresses (Kofer et al., 1998; Endo et al., 1999; Horvath et al., 2000) based on studies of different *ndh* disruptants. Significant accumulation of NADPH was observed in cyanobacterium *Synechocystis* PCC 6803 *ndh*B disruptant (Mi et al., 2000). Takabayashi et al. (2002) reported that *ndh*CKJ operon disruptants, Δ*ndh*KJ (same disruptant as in this study), Δ*ndh*CKJ, and *ndh*B, showed overreduction when post-illumination levels of steady-state fluorescence was analyzed. Under present condition, the rise of *F*_o_ at the elevated temperature was not attributed to the detachment of LHC II from PS II complex and the inactivation of PS II photochemical reaction (Schreiber and Armond, 1978), because the photochemical efficiency of PS II (*F*_v_*/F*_m_) did not significantly decrease after exposing WT and Δ*ndh*KJ or Δ*ndhC*KJ to 50°C (Yao et al., 2001; **Figure 1C**). The lower temperature-threshold for *F*_o_ raising (**Figure 1**) and the increase in *F*_o_ adapted at elevated temperature in Δ*ndhC*KJ (**Figure 2A**) may be related to the over reduction of the intersystem chain caused by over reduction of stroma. Accumulation of photoreductants, such as NADPH in stroma, likely generates active oxygen species that damage photosynthetic apparatus (Asada, 1999). Based on that the expression of *ndh*A was stimulated under oxidative stress condition, it was suggested that NDH functions in protecting plants against oxidative stress (Martin et al., 1996), and confirmed by the observation of remarkable accumulation of H_2_O_2_ in Δ*ndh*CKJ (Wang et al., 2006).

It has been demonstrated that PTOX is able to transfer electrons from PQ to oxygen without generating ROS (Cournac et al., 2000; Josse et al., 2003). NDH and PTOX involved in chlororespiration were suggested to provide and remove electrons, respectively, thus to balance the redox state of electron transporters (Niyogi, 2000; Martin et al., 2004; Streb et al., 2005). The slower increase in *F*_o_ after the temperature-threshold and the lower maximal level of *F*_o_ induced by high temperature in the NDH mutants (**Figures 1A,B**) suggests the contribution of electron donation from chlororespiration to PQ pool when tobacco plants response to the increased temperature. The significant decrease in *F*_v_*/F*_m_ in WT (**Figure 1C**) above 55°C might be attributive to the increased *F*_o_ probably causing by chlororespiration. The obvious increase of *F*_o_ in WT in the presence of PTOX inhibitor *n*-propyl gallate after adaptation at 50°C for 6 h (**Figure 2A**) indicates the operation of chlororespiration at the elevated temperature. By contrast, the similar effect of the PTOX inhibitor *n*-propyl gallate on *F*_o_ at 20°C in both WT and Δ*ndhC*KJ (**Figure 2A**) suggest that chlororespiration pathway has no obvious function under optimal physiological conditions. Although the decrease in *F*_v_*/F*_m_ in WT and Δ*ndhC*KJ was similar but the decrease was more pronounce in the presence of *n*-propyl gallate in the mutant (**Figure 2B**). The result suggests that suppression of both NDH and chlororespiration pathways causes the increased sensitivity to heat stress.

Changes in the redox state of intersystem electron carriers caused by chlororespiration have been suggested to tightly control the rate of PSI-driven cyclic electron flow *in vivo* (Joet et al., 2002a). Based on the data in **Table 1**, we concluded that NDH complex is involved in the heat-stimulated cyclic electron flow and chlororespiration in tobacco. The acceleration in NDH-mediated chlororespiration is related to the stimulation of NdhK and PTOX expressions under the high temperature stress conditions (**Figure 4**). Compared with the oxidation of P700, the difference between WT and *ndh*JK mutant in the re-reduction of P700^+^ under heat stressed conditions was much less significant (**Table 1**), suggesting a significantly promotion of cyclic electron flow in light, as well as the competition for electrons derived from PQ between PTOX in the chlororespiration pathway and P700^+^ post FR treatment. Therefore, the chlororespiratory pathway might play more important role in the dark while the cyclic electron flow around PS I might primarily function in photoprotection. Although the impairment of NDH activity in Δ*ndh*JK did not result in significant change in PTOX content under optimal conditions, the up-regulation level was much lower in Δ*ndh*JK than in WT (**Figure 4**), suggesting that NDH and PTOX must be kept in balance. The data in **Table 1** and **Figure 4** demonstrated that chlororespiration also increased to a certain extent in Δ*ndh*JK under heat stressed conditions, however, over-reduction of the intersystem chain was also observed, indicating an important role for NDH in photoprotection. The attenuated up-regulation of PTOX in Δ*ndh*JK likely resulted in weakened capacity of heat dispassion, causing the inactivation of enzymes for CO_2_ assimilation, such as Rubisco activase (**Figure 4**), thus the decrease of photosynthetic efficiency (**Figure 3**). Our results suggest that the cyclic electron flow around PS I and chlororespiration are coordinated to alleviate photoinhibition during heat stress.

## Ethics Statement

All the authors listed declare that the work described was original research that has not been published previously, and not under consideration for publication elsewhere without the written consent of the copyright-holder. All the authors listed have approved the publication of the research.

## Author Contributions

All authors listed, have made substantial, direct and intellectual contribution to the work, and approved it for publication. QL and Z-JY performed the research. QL revised the manuscript. HM designed the research and wrote the article.

## Conflict of Interest Statement

The authors declare that the research was conducted in the absence of any commercial or financial relationships that could be construed as a potential conflict of interest.

## Abbreviations

*F*_o_: minimum fluorescence yield at open PS II center
FR: far-red light
NDH: NAD(P)H dehydrogenase
PQ: plastoquinone
PS I: photosystem I
PS II: photosystem II
PTOX: plastid terminal oxidase
P700: reaction center chlorophyll of PS I
